# Selected summaries

**Published:** 2008

**Authors:** Sachin Talwar, Shiv Kumar Choudhary, Balram Airan

**Affiliations:** Department of Cardiothoracic and Vascular Surgery, All India Institute of Medical Sciences, New Delhi, India

## BIDIRECTIONAL GLENN AND ANTEGRADE PULMONARY BLOOD FLOW: TEMPORARY OR DEFINITIVE PALLIATION?

**Calvaruso DF, Rubino A, Ocello S, Salviato N, Guardí D, Petruccelli DF, Cipriani A, Fattouch K, Agati S, Mignosa C, Zannini L, and Marcelletti CF. Ann. Thorac. Surg.,2008;85:1389–1396.**

Marcellleti *et al*., who pioneered the extra cardiac Fontan (ECF) operation have added another dimension to the staged palliation of patients with a structurally and/or functionally univentricular heart (UVH). In this paper, the authors present their results of the bidirectional Glenn (BDG) shunt with preservation of antegrade pulmonary blood flow (APBF) in 246 patients recruited from three institutions over an 8-year period. Mean age and weight of the patients were 4.7 ± 6.2 years and 12.1 ± 5.4 kg, respectively. All the patients had restricted, but not critically reduced pulmonary blood flow. The patients who had undergone prior systemic to pulmonary artery shunts (BTS), right ventricle to pulmonary artery conduit, and the Norwood stage-I operation for hypoplastic left heart syndrome were excluded from this study. The common diagnoses were tricuspid atresia and double inlet or outlet types of UVH, which is quite similar to patients in the Indian subcontinent. Though there were no early deaths, there was a very high incidence of pericardial effusion (12.1%), prolonged pleural effusions (8.1%), and superior vena caval syndrome (9.7%). The latter was managed by reopening the azygous vein. The follow-up was available in all the patients over a mean period of 4.2 ± 2.8 years. During the follow up, patients underwent clinical examination, pulse oximetry, and echocardiogram. The systemic arterial saturation on room air were 81 ± 3.2%, 89 ± 3.7%, and 80 ± 7.2% preoperatively, at hospital discharge, and at the time of the last follow up, respectively. Actuarial freedom from completion ECF was 80% at five years and 70% at seven years. One hundred and seventy-three patients were well palliated with a mean systemic arterial oxygen saturation of 87 ± 4% on room air. Only 73 (29.7%) patients required completion to ECF for worsening cyanosis, fatigability on exertion, or increasing hematocrit. There were no late deaths, except in three patients who underwent ECF completion. The authors concluded that BDG with APBF is the surgery of choice for those with UVH, especially if the Fontan procedure is considered as a high-risk palliation, since the former can be accomplished with zero mortality. The claimed advantages of this approach over the traditional BDG with no APBF are lower mortality, favorable effects on cardiac function, prevention of arterio-venous fistulae, and better growth of pulmonary arteries. However, the results of this study must be interpreted with caution as there was no follow-up cardiac catheterization done to document the last two advantages. Also, a note must be made of a very high incidence of superior vena caval syndrome. The authors feel that this was not due to technical issues, but was probably related to the ‘high-risk category’ patients such as those with elevated pulmonary pressures and elevated pulmonary vascular resistance, significant atrioventricular valve regurgitation, or major aortopulmonary collaterals. They did not remodulate pulmonary blood flow after the appearance of superior vena caval syndrome, but, merely opened the azygous vein in all the patients. In fact, they recommend not to ligate the azygous vein while doing a BDG in small children less than 4 months of age. They think that it is very safe to leave the azygous vein open; but in due course of time, desaturation may set in necessitating conversion to Fontan.

## MODIFIED BLALOCK-TAUSSIG SHUNT: IMMEDIATE AND SHORT-TERM FOLLOW-UP RESULTS IN NEONATES

**Ahmad U, Fatimi SH, Naqvi I, Atiq M, Moizuddin SS, Sheikh KB, Shahbuddin S, Naseem TM, Javed MA. Heart Lung Circ. 2008;17:54–8.**

In this paper from Pakistan, Ahmad *et al*., present the immediate and short-term results of 22 patients, who underwent the modified Blalock-Taussig shunt (BTS) during the neonatal period. Mean age and weight were 11.2 ± 6.9 days and 2.9 ± 2.2 kg, respectively. The most common indications for the neonatal BTS were pulmonary atresia with intact ventricular septum (PA-IVS, 27.3%), and tricuspid atresia with right ventricular hypoplasia (22.7%). Prostaglandin E1 infusion was used in all the patients to maintain ductal patency for pre-operative optimization of oxygen saturation and the metabolic profile. Contrary to the Western world where majority of the BTS are now performed via a mid-line sternotomy, a posterolateral thoracotomy approach was used. All neonates received a 4 mm Gore-Tex graft as smaller sizes were not available at the authors’ institution. The ductus arteriosus was left open and was observed to close spontaneously on serial echocardiograms after stopping the prostaglandin E1 infusion. All patients received low-dose heparin in the immediate postoperative period which was later changed to low--dose aspirin. There were three early deaths, – one each due to shunt failure, malrotation of gut, and sepsis. The most common complications were atelectasis (22.7%), pneumonia (27%), sepsis (22.7%), and shunt thrombosis (22.7%). Two of the patients with shunt thrombosis died and two underwent revision of the shunt. Mean ventilation time was 3.9 ± 4.5 days and the mean ICU stay was 5.9 ± 4.5 days. Median follow-up was 18 months and there were 3 deaths after 4, 9, and 18 months due to unrelated causes. Two patients required shunt revision at 9 and 20 months after the first operation. Eleven patients underwent successful corrective surgery after a mean follow-up of 20 months. The results of this study indicate that BTS can be performed in the neonatal period with acceptable results. The patient population is quite similar in India and Pakistan due to prevalence of similar socioeconomic conditions and healthcare services. The patient age, weight, and size of the shunt did not correlate with early mortality and follow-up results. Among all cardiac defects, the outcome was the poorest for PA-IVS. Similar studies have been reported in the past from this region and the results have been identical, the only difference being a smoother postoperative course with the use of smaller shunts.

## THE OUTCOMES OF OPERATIONS FOR 539 PATIENTS WITH EBSTEIN'S ANOMALY

**Brown ML, Dearani JA, Danielson GK, Cetta F, Connolly HM, Warnes CA, Li Z, Hodge DO, Driscoll DJ. J Thorac Cardiovasc Surg 2008;135:1120–1136.**

This is the largest and the most comprehensive analysis of the experience with the surgical management of Ebstein's anomaly (EA) at the Mayo Clinic over 34-years (1972–2006) period. Five hundred and thirty-nine patients (8 days to 79 years; 24.1 ± 18.1 years) with EA underwent surgery for uncontrolled symptoms despite optimal medical therapy, presence of associated lesions (atrial or ventricular septa defects, pulmonary stenosis), and progressive cardiomegaly with reduced right ventricular (RV) or left ventricular (LV) function. Surgical procedures were developed by the authors themselves and included tricuspid valve repair (TVRep, *n* = 182, 33.8%), tricuspid valve replacement (TVR, *n* = 337, 62.5%), plication of the atrialized RV (*n* = 186, 34.5%), right atrial reduction plasty (*n* = 400, 74.2%), atrial septal defect closure (*n* = 450, 83.5%), and addition of a bidirectional Glenn shunt in a few patients when the RV was markedly dilated and dysfunctional. Anti-arrhythmia procedures such as division of accessory conduction pathways (*n* = 63, 11.6%), cryoablation of the fast pathway of the AV node (*n* = 10, 1.9%), or right sided Maze (*n* = 52, 9.7%) were performed in addition. The overall mortality was 5.9% for the entire cohort; it dropped to 2.7% after the year 2001. The 30-day and 1-, 5-, 10-, 15-, and 20-year survival rates were 94%, 92%, 88%, 85%, 81%, and 71%, respectively. On multivariate analysis, increased hematocrit, pulmonary valve stenosis, TVRep, presence of arrhythmias, hypoplastic pulmonary arteries, significant mitral valve regurgitation, and moderate to severe RV dysfunction were associated with higher mortality. For the TVRep group, the survival rates for 30-days and 1-, 5-, 10-, 15-, and 20-years were 95%, 93%, 93%, 88%, 87%, and 76%, respectively; whereas for the TVR group, the survival rates were 94%, 91%, 86%, 83%, 74%, and 68%, respectively. For the TVRep group, the survival rates of freedom from reoperation on the TV at 10-, 15-, and 20-years were 89%, 77% and 64%, respectively. For the TVR group, it was 83%, 70% and 59%. There was no difference in the reoperation-free actuarial survival between those with TVRep or TVR for patients 12 years of age or older, but there was a definite survival advantage in patients younger than 12 years of age who underwent TVRep. The authors used echocardiography to select patients for repair or replacement and MRI lately to quantify the RV function more accurately. The authors have, however, not specified their protocol which is used to select patients for surgery based on their MRI findings. The data from this study clearly indicates that patients with TVR had more severe disease than those with TVRep. In addition, significant TV regurgitation persisted in 33% patients with TVRep, but only in 1% with TVR. The authors recommend that the TV should be repaired if there is no more than moderate tricuspid regurgitation (TR) at the end of operation. However, for patients below 12 years of age greater degrees of TR may be accepted because of the need for reoperation, irrespective of whether they undergo repair or replacement. The detailed statistical analysis of more than 125 variables in over 500 patients makes this paper a landmark publication and helps in better understanding of the long-term outcome of this difficult subset of patients. However, many issues related to quantification of right ventricular dysfunction, the best modality of treatment, and precise protocol for arriving at this modality of treatment have not been addressed in this paper.

## SINGLE-STAGE VERSUS 2-STAGE REPAIR OF CO-ARCTATION OF THE AORTA WITH VENTRICULAR SEPTAL DEFECT

**Walters HL, Ionan CE, Thomas RL, Delius RE. J Thorac Cardiovasc Surg 2008;135:754–6.**

The decision-making process for surgical repair of co-arctation of the aorta (CoA) and ventricular septal defect (VSD) is difficult as one may perform either a complete one-stage repair or do it in two stages with the initial stage being repair of the CoA with or without a pulmonary artery banding. This retrospective study from the Children's hospital of Michigan, US, attempts to address this issue. Out of the 46 patients operated at this institution, 23 underwent single-stage repair and 23 underwent two-stage repair. The cardiac morphology and the weight of the patients were comparable in both groups. There was one early death in each group. Postoperative complications were similar, but seven patients in the single-stage group had delayed sternal closure to tide over the initial period of hemodynamic instability. The follow-up for the entire group ranged between 3.3–6.6 years. The actuarial survival was 95.7% in single-stage group vs. 90.6% in two-stage group. The freedom from all types of cardiac reintervention was 89.8% in single-stage group and 84.9% in two-stage group. The authors concluded that the advantages of single-stage repair over the two-stage repair for CoA-VSD are earlier age at completion of repair, fewer re-operations, fewer incisions, avoidance of complications related to pulmonary artery banding, and the ability to correct the entire range of aortic arch hypoplasia from the single-stage midline approach. However, the results of this strategy are to be interpreted with caution because the study spans over two different periods and the patients in the earlier part of the study had the two-stage approach. This shift from the two-stage to one-stage repair is possible only with improvements in neonatal cardiopulmonary bypass, refinements of myocardial protection techniques, and better intensive care. In developing countries, this may still not be applicable, particularly if the patients need to be in the intensive care unit with open sternum for a prolonged period.

